# Comparison of the Conjunct Effects of Electrical Stimulation and Whole-Body Vibration Therapy with Transcranial Direct Current Stimulation and Whole-body Vibration Therapy on Balance and Function in Children With Spastic Cerebral Palsy

**DOI:** 10.7759/cureus.61511

**Published:** 2024-06-01

**Authors:** Zainab Hassan, Mohammad-Reza Hadian, Syed ali Hussain, Azadeh Shadmehr, Saeed Talebian, Hossein Bagheri, S. Mohsen Mir, Syed Asadullah Arslan

**Affiliations:** 1 School of Rehabilitation, Tehran University of Medical Sciences, Tehran, IRN; 2 Brain and Spinal Cord Injury Research Center, Institute of Neuroscience, Tehran, IRN; 3 Department of Physical Therapy, Riphah International University, Lahore, PAK

**Keywords:** whole body vibration, functional electrical stimulation, transcranial direct current stimulation, spasticity, muscle strength, lower extremity, grip strength, cerebral palsy, balance

## Abstract

Background and objectives: Cerebral palsy is a neurodevelopmental condition that results in impaired movement and posture, often accompanied by disturbances in balance and functional abilities. Recent advances in neurorehabilitation, including whole-body vibration therapy (WBVT), functional electrical stimulation, and transcranial direct current stimulation, show promise in enhancing traditional interventions and fostering neuroplasticity. However, the efficacy of their conjunct effects remains largely uncharted territory and warrants further exploration. The objective of the study was to compare the conjunct effects of functional electrical stimulation (FES) and WBVT with transcranial direct current stimulation (tDCS) and WBVT on lower extremity range of motion (ROM), dynamic balance, functional mobility, isometric muscle strength and hand grip strength in children with spastic cerebral palsy.

Methods: A randomized clinical trial was carried out on 42 children of both genders with spastic cerebral palsy, aged 5-15 years. The children were divided at random into three groups (14 in each group). In Group A, there were three (21.42%) males and 11 (78.57%) females, in Group B, eight (57.14%) were males and six (42.85%) were females, and in Group C, six (42.85%) children were males and eight (57.14%) were females. Group A received WBVT only, Group B received WBVT and FES, and Group C received WBVT and tDCS. The intervention was applied four times a week for four consecutive weeks. The data was collected two times before and immediately after four weeks of intervention. Lower extremity ROM was measured by a goniometer, functional mobility or dynamic balance was measured by a Time Up and Go test, isometric muscle strength was measured by a digital force gauge, and hand grip strength was assessed by a digital hand-held dynamometer. IBM SPSS Statistics for Windows, Version 27.0 (Released 2020; IBM Corp., Armonk, New York, United States) was utilized for statistical analysis.

Results: The mean age of the children in groups A, B, and C was 12.21±2.11 years, 11.71±2.01, and 11.07±2.01 years respectively. Intergroup analysis revealed a statistically significant difference (p<0.05) in the lower extremity range of motion, and functional mobility. Hand grip strength and isometric muscle strength between three groups. Post hoc analysis revealed that WBVT with transcranial direct current stimulation combined showed the most improvement.

Conclusion: The study concluded that positive effects were seen in all three groups but tDCS with WBVT was found to be most effective in improving lower extremity ROM, functional mobility or dynamic balance, isometric muscle strength, and hand grip strength in children with spastic CP. The differences between the groups were statistically significant. The effect size was substantial enough to surpass established clinical benchmarks, indicating that the observed improvements are likely to have meaningful and beneficial impacts on patient outcomes.

## Introduction

Cerebral palsy (CP) presents a complex neurodevelopmental challenge and encompasses a broad spectrum of conditions, impacting not only physical abilities but also cognitive function and daily functioning. It is a prevalent physical impairment in the initial days of life, occurring in 2-2.5 cases per 1000 live births [1]. The condition of CP can be grouped into three main types: spastic CP, dyskinetic CP, and ataxic CP. Spastic diplegia is among the most prevalent kind of CP diagnosed across the world. Muscular stiffness, particularly in the lower limbs, is frequent in the disease. It shows a symmetric relationship between the lower and upper extremities. Children with spastic CP commonly exhibit challenges with motor coordination, spasticity, and balancing, which contribute partly to gait impairments [1].

The diagnosis and management of CP continue to pose significant challenges within the medical community, despite advances in understanding and treatment modalities. Typically identified through clinical assessment within the first or second year of life, CP manifests through a spectrum of characteristic findings, including spasticity, poor coordination, cognitive impairments, epilepsy, and behavioral issues. Of particular note is the observation of poor proprioception among children with CP, complicating their motor function and coordination [2].

In recent years, research efforts have focused on refining therapeutic approaches and exploring innovative interventions to enhance the efficacy of CP management. Novel interventions such as constraint-induced movement therapy (CIMT), which is a type of rehabilitation treatment that helps patients, restore upper limb function through increasing the usage of the affected or impaired upper extremity, virtual reality-based rehabilitation, which includes moving and completing tasks in a virtual world and can enhance motor skills and robotics-assisted therapy which are devices or gadgets that employ sensors to detect human movement and orientation, and then use that input to interact, hold promise in addressing specific motor impairments and promoting neuroplasticity in children with CP [3]. Additionally, advances in assistive technology, such as exoskeletons and functional electrical stimulation (FES) devices, and transcranial DC stimulation offer new avenues for improving mobility and independence among individuals with CP [4].

During the past few years, whole-body vibration therapy (WBVT) has been believed a promising treatment modality for the management of CP. WBVT is an innovative intervention that entails the exposure of the entire body to mechanical vibrations, which elicit reflex muscle contractions and stimulate proprioceptive feedback mechanisms. By targeting sensory-motor integration and neuromuscular coordination, WBVT holds promise in enhancing balance, motor functioning, and gait in children with CP [5]. Cheng et al. revealed that WBVT of two months restored the tone of the muscles, increased active joint mobility, and boosted ambulatory activity in kids suffering from CP for a minimum of three days [6]. It has been hypothesized that introducing the WBVT across multiple configurations, including weight- as well as non-weight-bearing stances involving both the lower and upper limbs, might promote brain plasticity via the cross-training action [7].

FES and transcranial direct current stimulation (tDCS) have emerged as promising modalities for augmenting motor control and facilitating neuroplasticity in individuals with CP. FES involves the application of electrical currents to specific muscles or nerves, aiming to induce muscle contractions and enhance functional actions. This technique holds the potential for addressing muscle weakness, spasticity, and impaired motor control in individuals with CP, offering a non-invasive and targeted intervention [8]. El-Shamy et al. determined the impacts of FES on postural control, three days per week for three months in addition to the regular physiotherapy program [9]. The results demonstrated a substantial increase in the postural stability of the treatment group.

Similarly, tDCS has garnered attention as a non-invasive neuromodulation technique for enhancing cortical excitability and promoting neuroplasticity in individuals with CP. It is a low-cost and low-risk intervention that may be readily delivered in a clinical setting or in homes to induce a considerable improvement in spasticity, balancing, motor functioning, and gait problems. tDCS controls neuronal activity and alters resting membrane potential by administering low-intensity direct current to the cerebral cortex via scalp surface electrodes. The electrode polarity is hypothesized to determine whether tDCS has an excitatory or inhibitory effect on cortical activity, with anodal stimulation enhancing cortical excitability and cathodal stimulation reducing excitability [10]. It is hypothesized that WBVT, when used in conjunction with tDCS, may increase activity in the motor cortex, which is critical for the rehabilitation of brain-injured patients [11]. Collange-Grecco et al. revealed that combining active tDCS and dual-task training improved functional mobility, as well as functionality and cognitive abilities, one month after the therapy ended [12].

Overall, WBVT, FES, and tDCS represent valuable adjuncts to traditional rehabilitation approaches in the management of CP. Previous research mostly reported the positive effects of WBVT on balance, strength, gait, walking speed, standing function, spasticity, flexibility, proprioception, and range of motion (ROM). But, to the best of our knowledge, there is no evidence found of WBVT in conjunction with FES and tDCS for spastic CP patients. The hypothesis suggests that WBVT's vibratory input, alongside tDCS's central stimulation, enhances function by modulating neuronal excitability and sensory information. So, the purpose of the present study was to compare the conjunct effects of FES and tDCS in conjunction with WBVT on lower extremity ROM, balance, functional mobility, isometric muscle strength, and hand grip strength in children with spastic CP and to provide evidence for more effective, low-cost, and feasible management techniques.

## Materials and methods

Study design and population

This double-blinded randomized clinical trial was conducted at the Dimension National Institute of Special Children and Sehat Medical Complex in Lahore, Pakistan, as a research thesis of Tehran University of Medical Sciences, Iran.. The duration of the study was from June 2023 to May 2024. The study was approved by the Research Ethics Committees of the School of Nursing and Midwifery & Rehabilitation, Tehran University of Medical Sciences (approval number: IR.TUMS.FNM.REC.1402.041) and the clinical trial was registered in the Iranian Clinical Trial Registry (registration number: IRCT20090301001722N29). Parents of all participants provided written consent, and inclusion in the study was entirely voluntary.

In this double-blinded study, care providers of children were blinded to their assigned groups while outcome assessors were blinded to the treatment regimens and research hypotheses. The treatment and assessments were carried out by different people. Two physical therapists, already working in the setting, participated in providing care and delivered the treatment protocol to children, shared by the primary investigators of the study.

Selection criteria

Participants underwent screening to determine eligibility, and a total of 42 children with spastic CP were selected according to the defined selection criteria. Participants were recruited through a simple random sampling technique.

The inclusion criteria of the study were children of both genders diagnosed with spastic CP, aged 5-15 years, and having spasticity scores of 1, 1+, and 2 on the modified Ashworth scale, as well as Gross Motor Function Measure level I and II with Manual Ability Classification System level I and II. Children with fixed musculoskeletal deformity, recent surgery (<1 year ago), unhealed fracture, epilepsy, auditory or visual problems, receiving botulinum toxin therapy, other forms of CP (ataxic, flaccid, and dyskinetic), and sensory loss in the upper and lower extremities were excluded. Parents/children who were not interested in finishing the course of treatments were also excluded.

After recruitment, all participants were assigned serial numbers (from 1-42) and divided into three groups (Groups A, B, and C). Randomization was carried out using Version 1.0 of Randomized Allocation Software (Department of Anesthesia at Isfahan University of Medical Sciences, Isfahan, Iran). A timetable was prepared for the interventional portion of the trial, and parents were asked to make sure that participants were present during that time to avoid missed sessions.

Interventions

WBVT was administered to Group A, FES with WBVT was given to Group B whereas Group C received WBVT with tDCS. Additionally, all participants received conventional physical therapy, including stretching exercises, parallel bar exercises, mat exercises, and balance board exercises for 15-20 minutes per day, as they had received before the study.

Children in Group A received WBVT intervention for 20 minutes each day, four days a week, for four weeks. The participants were asked to stand on the vibration platform. Children were standing barefoot with their feet parallel and slightly flexed (30°). When standing, the feet were spaced evenly apart from the device's center line. During the 20-minute treatment session, three minutes of vibration training and two minutes of rest were repeated four times. The frequency used for WBVT was 18 Hz with an amplitude of 12 mm and slowly increased by 1 Hz every two seconds until reaching 18 Hz. The WBVT protocol remained the same for all groups; therefore, in Group B, FES with rectangular biphasic pulses with a pulse width of 250 μs, a stimulus intensity of 70 mA, and a frequency ranging from 40 Hz was applied for four days a week for four weeks on motor points of the spastic muscles mainly for the lower extremity (quadriceps and gastrocnemius and soleus). Furthermore, Group C received tDCS with 2 mA intensity for 20 minutes a day, four days a week for four weeks by using 5x7 cm^2^ electrodes. Anodal tDCS was placed over the primary motor cortex (area concerned with the lower extremity function) and cathodal tDCS was placed over the contralateral supraorbital region. A total of 16 treatment sessions were given, four days a week for four consecutive weeks.

**Figure 1:**
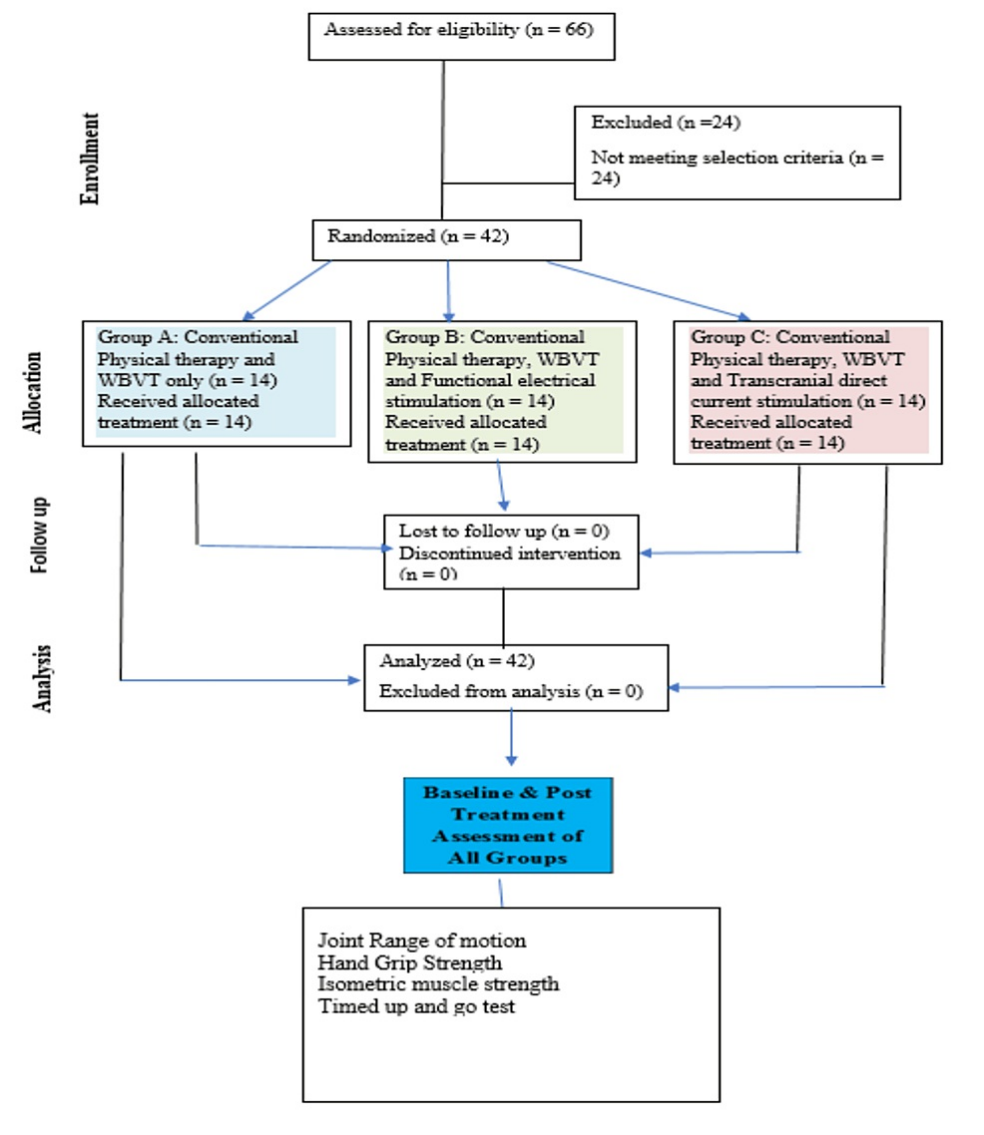
CONSORT diagram CONSORT: Consolidated Standards of Reporting Trials; WBVT: whole-body vibration therapy

Figure 1 shows the Consolidated Standards of Reporting Trials (CONSORT) flow diagram.

Outcome measures

Lower extremity joint ROM (hip, knee, and ankle) was assessed using a universal goniometer. The test and retest reliability of a universal goniometer had an intraclass correlation coefficient (ICC) of 79-92% [13].

Hand grip strength was assessed with a Camry digital handheld dynamometer (Camry Industries Company Ltd., Kowloon, Hong Kong). Grip strength is an estimation of skeletal muscle power or the greatest force or tension produced by an individual's forearm muscles. It may be employed as a screening tool to assess upper body strength. The subject was instructed to press the dynamometer maximum for three seconds while maintaining a neutral upper-extremity stance. One trial was done until the participants learned before the actual reading was measured. The hand grip strength was measured for both hands (affected side and normal side) before and after the intervention. The dynamometer handle's opening was calibrated to the size of the individual's hand, and the readings were obtained with the subject in a standing posture, with arms parallel to the body but not directly in contact with it, shoulder adducted, and a neutral forearm. The test and retest reliability of a digital hand-held dynamometer had an ICC of 80-94% [14].

Isometric muscle strength (hip extensors, hip adductors, knee extensors, knee flexors, and ankle plantar flexors) was measured using a digital force gauge SF-500 in kg. It had different attachments. ICC coefficients for all measures were 75% or above [15].

Functional mobility was assessed using a timed-up-and-go test. The children were seated on a standard armchair and instructed to get up and walk; the participants had to stand up from the chair, walk to a line on the floor 3 meters away, turn around, return to the chair, and sit down again. The time required to complete the task was recorded by using a stopwatch. One practice session was performed once before the actual test. Test-retest reliability of timed-up-and-go had an ICC of 87-99% [16].

All participants were assessed before the intervention and immediately after the four weeks (16 sessions) of therapy. There were no dropouts in the study. There were no protocol violations in the study, and an intention-to-treat analysis was performed, as all participants were analyzed according to the groups to which they were originally assigned.

Statistical analysis

Mean and standard deviation (SD) of the demographic data were calculated. The normality of the data was checked and the Shapiro-Wilk test was applied. The mean, median, and SD were calculated for all the outcome measures pre- and post-intervention. As the data were normally distributed, one-way analysis of variance (ANOVA) was used to determine whether there were any statistically significant differences between the means of the three groups. The post hoc test was applied to find the most effective group with a confidence interval of 95%, using a 5% significance level. IBM SPSS Statistics for Windows, Version 27.0 (Released 2020; IBM Corp., Armonk, New York, United States) was used for data analysis.

## Results

Table 1 shows the demographic statistics of the study. The mean age of the children in groups A, B, and C was 12.21±2.11, 11.71±2.01, and 11.07±2.01, respectively. In Group A, 21.42% were males and 78.57% were females. In Group B, 57.14% were males and 42.85% were females, and in Group C, 42.85% of the children were males and 57.14% were females. The mean BMI of the children in groups A, B, and C was 23.2±7.05, 20.1±5.53, and 19.6±3.90, respectively. Among 42 participants, the right side was affected in 20 children and the left side was affected in 22 children.

**Table 1:** Demographic statistics WBVT: whole-body vibration therapy; tDCS: transcranial direct current stimulation; FES: functional electrical stimulation

Groups	N	Male, n (%)	Female, n (%)	Age (years), mean±SD	p-value	BMI (*kg/m^2^)*, mean±SD
Group A (WBVT only)	14	3 (21.42)	11 (78.57)	12.21±2.11	.346	23.2±7.05
Group B (WBVT and FES)	14	8 (57.14)	6 (42.85)	11.71±2.01		20.1±5.53
Group C (WBVT and tDCS)	14	6 (42.85)	8 (57.14)	11.07±2.01		19.6±3.90

**Figure 2:**
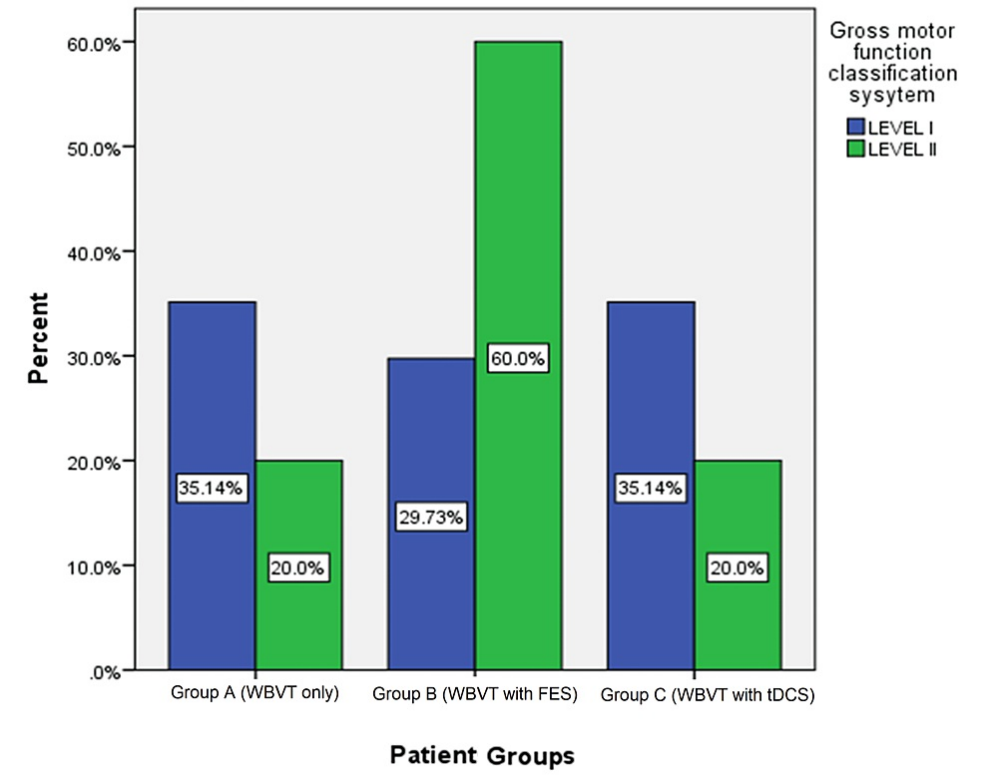
Comparison of gross motor classification system levels in the three groups WBVT: whole-body vibration therapy; FES: functional electrical stimulation; tDCS: transcranial direct current stimulation

Figure 2 depicts the percentage of the Gross Motor Classification System level of participants in each group. Of the participants in Group A, 35.14% were at Level I whereas 20% were at Level II. In Group B, 29.73% were at Level I whereas 60% were at Level II. Finally, in Group C, 35.14% were at Level I and 20% were at Level II.

**Figure 3:**
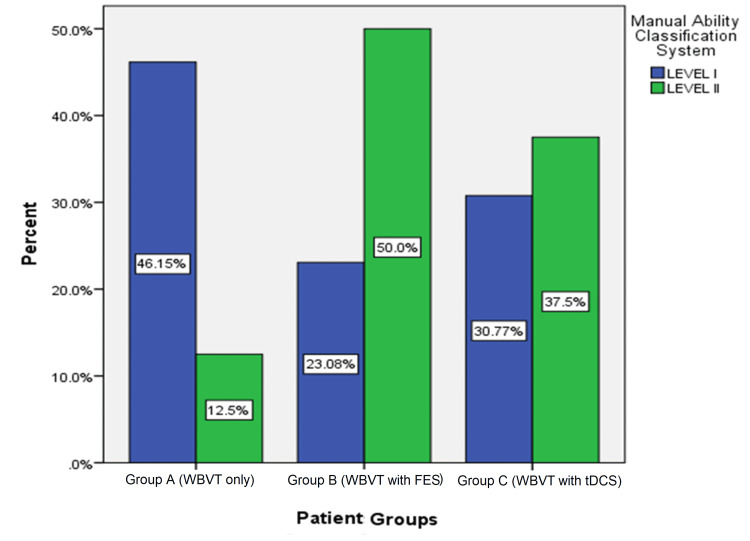
Comparison of manual ability classification system levels in the three groups WBVT: whole-body vibration therapy; FES: functional electrical stimulation; tDCS: transcranial direct current stimulation

Figure 3 depicts the percentage of the Manual Ability Classification System level of participants in each group. Of the participants in Group A, 46.14% were at Level I whereas 12.5% were at Level II. In Group B, 23.08% were at Level I whereas 50% was at Level II. Finally, in Group C, 30.77% were at Level I and 37.5% were at Level II.

Table 2 displays the ROM for the hip, knee, and ankle joints. The one-way ANOVA revealed p-values higher than 0.05 for hip flexion, extension, abduction, adduction, both internal and external rotation, knee flexion and extension, and ankle plantar flexion and dorsiflexion before treatment, indicating that there was no statistically significant difference between groups. The post-treatment p-value was below 0.05, indicating a statistically significant difference in improvement in hip flexion, extension, abduction, adduction, internal and external rotation, knee flexion and extension, and ankle plantar flexion and dorsiflexion between the groups. The post hoc analysis revealed that Group C showed the most significant improvement among all three groups.

**Table 2:** Range of motion of hip, knee, and ankle (intergroup analysis) * shows significant difference between groups on the post-hoc test.

Joint/Range			Group A, mean ± SD		Group B, mean ± SD	Group C, mean ± SD	p-value
Hip	Flexion	Pre-treatment	68.28 ± 12.88	65.85 ± 13.78		73.14 ± 12.88	.341
		Post-treatment	72.35 ± 12.97	71.21± 14.44		85.92 ± 12.55*	.009
	Extension	Pre-treatment	6.28 ± 2.23	5.71 ± 1.89		6.57 ± 1.60	.495
		Pre-treatment	8.57 ± 2.34	9.28 ± 2.12		16.00 ± 2.77*	.001
	Adduction	Post-treatment	8.42 ± 1.78	9.35 ± 1.86		9.64 ± 2.67	.305
		Pre-treatment	14.35 ± 1.94	16.07 ± 2.46		21.07 ± 2.97*	.001
	Abduction	Post-treatment	16.71 ± 3.60	16.14 ± 3.23		17.64 ± 3.10	.489
		Pre-treatment	20.14 ± 3.59	21.00 ± 2.93		28.42 ± 3.56*	.001
	Internal Rotation	Post-treatment	19.85 ± 5.18	20.21 ± 6.63		22.57 ± 6.23	.443
		Pre-treatment	22.71 ± 5.36	24.21 ± 6.67		34.14 ± 6.63*	.001
	External Rotation	Post-treatment	22.50 ± 5.41	23.14 ± 6.45		23.92 ± 6.20	.823
		Pre-treatment	26.35 ± 5.25	29.28 ± 6.43		36.00 ± 6.17*	.001
Knee	Flexion	Post-treatment	62.35 ± 10.26	61.21 ± 15.46		63.28 ± 9.94	.903
		Pre-treatment	72.00 ± 11.34	74.21 ± 13.00		84.28 ± 10.78*	.025
	Extension	Post-treatment	-8.21 ± 2.22	-8.14 ± 1.99		-7.64 ± 2.13	.741
		Pre-treatment	-7.21 ± 1.42	-6.07 ± 1.54		-2.42 ± 1.82*	.001
Ankle	Plantar Flexion	Post-treatment	31.71 ± 5.64	32.14 ± 5.54		34.64 ± 4.71	.301
		Pre-treatment	35.50 ± 5.09	37.64 ± 5.59		45.42 ± 4.56*	.001
	Dorsi Flexion	Post-treatment	10.42 ± 3.27	12.00 ± 2.14		10.64 ± 2.61	.265
		Pre-treatment	12.71 ± 3.09	15.57 ± 2.79		19.42 ± 2.73*	.001

Table 3 displays the results of the timed-up-and-go score. The one-way ANOVA showed the p-value for pre-treatment was 0.673, which shows that there was no statistically significant disparity between the groups. For post-treatment, the p-value was 0.005, showing a statistically significant disparity in improvement in functional mobility between the groups. The post hoc analysis revealed that Group C showed the most significant improvement among all three groups.

**Table 3:** Timed-up-and-go test (intergroup analysis) * shows significant difference between groups on the post-hoc test

Outcome Variable	Groups	N	Pre-Treatment Values (seconds)			Post-Treatment Values (Seconds)		
			Mean	S.D.	p-value	Mean	S.D.	p-value
Timed up and go test score	Group A	14	16.14	4.36	.673	13.58	4.52	.005
	Group B	14	16.63	4.82		13.06	4.86	
	Group C	14	15.21	3.47		8.45*	3.14	

Table 4 displays the results of isometric muscle strength. The one-way ANOVA showed that the p-value of hip extensors and adductors, knee flexors and extensors, and ankle plantar flexors for pre-treatment was higher than 0.05 indicating there was no statistically significant disparity between the groups. For post-treatment, the p-value was lower than 0.05 indicating that there was a statistically significant disparity in improvement in isometric muscle strength between the groups. The post hoc analysis showed that Group C showed the most significant improvement among all three groups.

**Table 4:** Isometric muscle strength (intergroup comparison) * shows significant difference between groups on the post-hoc test

Joint			Group A, mean ± SD (Kg)	Group B, mean ± SD (Kg)	Group C, mean ± SD (Kg)	p-value
Hip	Extensors	Pre-treatment	6.87 ± 1.29	5.76 ± 1.30	6.43 ± 1.60	.125
		Post-treatment	9.42 ± 1.46	9.36 ± 1.80	12.38 ± 2.04	.001
	Adductors	Pre-treatment	6.19 ± 1.13	6.73 ± 1.57	6.05± 1.55	.424
		Post-treatment	9.21 ± 1.29	10.52 ± 1.93	11.22 ± 2.35	.027
Knee	Knee extensors	Pre-treatment	9.59 ± 1.32	10.36 ± 1.44	9.63 ± 1.27*	.256
		Post-treatment	12.89 ± 1.55	14.71 ± 1.70	15.22 ± 2.18	.004
	Knee flexors	Pre-treatment	7.95 ± 0.95	7.79 ± 1.47	7.92 ± 1.67	.949
		Post-treatment	12.18 ± 1.28	13.31 ± 1.78	15.08 ± 1.95*	.001
Ankle	Plantar flexors	Pre-treatment	7.06 ± 1.01	7.66 ± 1.45	7.82 ± 1.19	.245
		Post-treatment	11.39 ± 1.58	13.41 ± 1.86	15.09 ± 2.02*	.001

Table 5 displays the results of hand grip strength. The one-way ANOVA showed the p-value for pre-treatment was 0.795 which shows that there was no statistically significant disparity among the groups. For post-treatment, the p-value was 0.008 meaning that there was a statistically significant disparity in improvement in grip strength between the groups. The post hoc analysis revealed that Group C showed the most significant improvement among all three groups.

**Table 5:** Hand grip strength (intergroup comparison) * shows significant difference between groups on the post hoc test

Groups	N	Pre-Treatment Values (Kg)			Post-Treatment Values (Kg)		
		Mean	S.D.	P-value	Mean	S.D.	P-value
Affected side							
Group A	14	7.41	2.30	.795	9.25	2.70	.008
Group B	14	7.66	1.38		11.0	2.51	
Group C	14	7.14	2.18		12.6*	2.97	
Normal side							
Group A	14	10.91	2.92	.406	9.25	2.70	.009
Group B	14	10.18	1.62		13.14	1.83	
Group C	14	9.77	2.01		15.82*	0.52	

## Discussion

The results of the present study showed that WBVT, FES with WBVT, and tDCS with WBVT were effective in improving the ROM of lower extremities, including hip flexion, extension, abduction, adduction, internal and external rotation, knee flexion and extension, and ankle plantar flexion and dorsiflexion, functional mobility, or dynamic balance, isometric muscle strength of hip extensors and adductors, knee flexors and extensors, and ankle flexor and hand grip strength. Interestingly, the conjunct effects of tDCS and WBV were found to be most effective in improving lower extremity ROM, functional mobility, isometric muscle strength, and hand grip strength in children with spastic CP.

In the present study, WBVT was provided to children with spastic CP for 20 minutes each day, four days a week, for four weeks. The WBVT stimulation frequency was 18 Hz and steadily increased by one Hz every two seconds until the target frequency was obtained. Vibrations are thought to cause the recruitment of muscles by loading the specified stretch-sensitive receptors (spindles) and consequently alpha motoneuron activities. Previous research showed that WBVT induces electro-myographic activity, comparable to traditional resistance training [17]. Therefore, WBVT may be used as a type of muscle strengthening that is primarily not dependent on the patient's motivation, in contrast to resistance training, which often requires great contribution from the participants [18].

El-Shamy et al. demonstrated that WBVT can enhance muscular strength and balance among children suffering from diplegic CP [19]. In the present study, our results showed that WBVT was seen to improve hip flexion (mean difference 4.07), extension (mean difference 2.29), abduction (mean difference 3.43), adduction (5.93), internal and external rotation (mean difference 2.86 and 3.85, respectively), knee flexion and extension (mean difference 9.65 and 1.00, respectively), and ankle plantar flexion (mean difference 3.79) and dorsiflexion (mean difference 2.29). Besides, dynamic balance (mean difference 2.56), isometric muscle strength of hip adductors and extensors, knee flexors and extensors, ankle planter flexors, and hand grip strength (mean difference 1.84) were seen. Similar to our findings, Cheng et al. found that WBVT can improve spasticity, ambulatory functioning, and active ROM [20]. Another previous study has shown that repeated use of WBVT could improve muscular strength, balance, and bone health over time [18].

In our study, WBVT alone improved functional mobility by 2.56 seconds, a combination of WBVT and FES improved functional mobility by 3.57 seconds, and a combination of WBVT and tDCS improved functional mobility by 6.76 seconds, as analyzed by the timed-up-and-go score. Ko et al. also reported the impact of WBVT on functional mobility and other parameters in 24 children with spastic CP [21]. After a three-week intervention, they discovered that the timed-up-and-go scores of the WBVT group improved significantly (p = 0.039) in comparison to the control group receiving conventional physiotherapy. A study by Lazzari et al. in 20 children with CP found that tDCS can enhance the impacts of virtual reality training on both static and functional balance [22]. They discovered a significant disparity in the timed-up-and-go test scores between the experimental (active tDCS+ virtual reality training) and control groups (sham tDCS+ virtual reality training) following a one-month intervention. Based on these studies, it is apparent that both tDCS and WBV can improve functional mobility. In the present study, the group that received the combined therapy of WBVT and tDCS demonstrated the most substantial enhancement in the timed-up-and-go score with a mean difference of 6.76 as compared to the other two groups.

In the present study, a statistically significant disparity was observed in pre- and post-intervention weeks by the combination of FES and WBV. It was found to be useful in improving lower extremity ROM, functional mobility and dynamic balance, isometric muscle strength, and hand grip strength in children with spastic CP. Although no study was found that saw combined effects of FES and WBV, a study by Sporea et al. supported the results by reporting the beneficial effects of FES in children with CP [23]. They discovered that FES proved to be an efficient method to boost motor function in terms of coordination, ROM, and grasp strength, evidently minimizing limitations in functioning and enhancing the ability to perform routine activities in children with spastic CP.

In the present study, the inter-group analysis showed a statistically significant disparity with post-treatment values for hand grip strength (p-value 0.008), isometric muscle strength of hip, knee, and ankle region (p<0.05), functional mobility (p-value 0.005), and hip, knee, and ankle ROM (p<0.05). The post hoc analysis revealed that Group C receiving the treatment of tDCS and WBV showed the most significant improvement among all three groups. However, there is no protocol in the literature similar to what we used for the assessment of the beneficial effects of conjunct tDCS and WBVT. There is a study available regarding the conjunct effect of tDCS with treadmill training, which showed improved static balance and functional capacity in children with CP [24]. In line with the present trial, Hikosaka et al. discovered that tDCS causes neuromodulation, which may lead to increased handgrip strength [25]. Another study by Salazar Fajardo et al. examined the combination of tDCS and neurodevelopmental treatment (NDT) [26]. Although their intervention protocol was different from the present study, they highlighted the conjunct effects of tDCS with NDT and proposed that tDCS coupled with NDT might be a potential solution for kids with CP since it can improve motor function and decrease spasticity in this demographic.

In the current study, tDCS was found to be effective in improving hip flexion, extension, abduction, adduction, internal and external rotation, knee flexion and extension, and ankle plantar flexion and dorsiflexion (p<0.05). Previous studies have revealed that tDCS improves passive joint ROM. Cathodal but not anodal or sham circumstances result in a considerable increase in ankle ROM [27].

Although Gillick et al. reported that tDCS paired with constraint-induced mobility therapy was effective, their study demonstrated no substantial difference in hand function between the treatments (active tDCS) and control (sham tDCS) groups, despite overall improvements [28]. Unlike the present investigation, one study with a limited sample size reported that tDCS did not affect hand grip strength [10]. This contradiction regarding the lack of an impact can be attributed to various factors such as a small sample size of eight patients. Also, only six of them performed the hand grip strength test; the other two patients were unable to perform the test because of severe hand impairment, which may have affected the power of the analysis and limited the generalizability of their findings. Another factor in their study was the variability in the severity and type of hand impairment in the patients, which may have influenced the results. Also, the lack of gain in grip strength can be attributed to the sensitivity of testing tools. It is most likely that the parameters used in the hand grip strength test were not finely tuned to identify any subtle changes caused by the intervention. Lastly, in their study, the tDCS was applied just once, which may be insufficient to cause significant changes in hand grip strength. Multiple tDCS sessions over time might be needed to achieve apparent effects on muscle strength [10].

Literature suggests that tDCS modulates brain activity in certain parts where electrodes tend to be positioned. It is applied through the motor cortex to alter the brain's motor activity. The neurophysiological impacts of anodal stimulation improve motor development by increasing cortical excitation. These gains help to improve the spasticity and static balance in CP patients [29]. WBVT has been shown to increase neural drive to the musculature; this heightened response promotes the activation of previously inactive motor units, hence improving muscular strength and mass in CP [30]. It was hypothesized that integrating central stimulation with peripheral nerve stimulation could minimize the time required to elicit strong excitability increases [11]. By implementing this combination, in our study, the conjunct effects of tDCS and WBV were found to be most effective in improving lower extremity range of motion, functional mobility and dynamic balance, isometric muscle strength, and hand grip strength in children with spastic CP.

Recommendations and limitations

Further studies are recommended with long-term follow-ups with the use of different combinations of parameters and electrode placement of tDCS. Therapists are encouraged to incorporate these interventions in clinical settings for the treatment of children suffering from spastic cerebral palsy. In our study, only children with spastic CP were recruited, so the findings may not be generalized to other types or populations. Moreover, a follow-up of only four weeks was taken, so it was not known whether the effects were maintained for the long term or not. 

## Conclusions

Positive effects were seen in all three groups (WBVT alone, FES with WBVT, and tDCS with WBVT) were found to be effective in improving hip flexion, extension, abduction, adduction, internal and external rotation, knee flexion and extension, ankle plantar flexion and dorsiflexion, functional mobility or dynamic balance, isometric muscle strength of hip extensors and adductors, knee flexors and extensors, ankle flexor, and hand grip strength. It is worth mentioning that the conjunct effects of tDCS and WBV were more pronounced and to be most effective in improving lower extremity ROM, functional mobility and dynamic balance, isometric muscle strength, and hand grip strength in children with spastic CP. The differences between the groups were statistically significant. The effect size was substantial enough to surpass established clinical benchmarks, indicating that the observed improvements are likely to have meaningful and beneficial impacts on patient outcomes.
